# Pharyngeal Mucosal Space Abscess: A Special Entity That Merits Special Management—Our Experience in 106 Cases

**DOI:** 10.3390/jcm14051515

**Published:** 2025-02-24

**Authors:** Charikleia Maiou, Vasileios A. Lachanas, Stergios Nik Doumas, Nikolaos Kalogritsas, Anna Mpouronikou, Jiannis Hajiioannou, Efthymia Petinaki, Charalampos E. Skoulakis

**Affiliations:** 1 Department of Otorhinolaryngology, University Hospital of Larissa, Viopolis, 41334 Larissa, Greece; 2 Maxillofacial Unit, University Hospital of Larissa, 41334 Larissa, Greece; 3 Department of Microbiology, University Hospital of Larissa, 41334 Larissa, Greece

**Keywords:** deep neck spaces, deep neck infections, pharyngeal mucosal space, abscess

## Abstract

**Background:** Deep neck infections represent a common but heterogeneous medical condition, as clinical manifestations and outcomes vary, depending, among others, on the infection site. The pharyngeal mucosal space (PMS) represents the most superficial neck space, confined between the pharyngeal mucosa and the pharyngeal constrictor muscles. The collection of pus in the PMS (pharyngeal mucosal abscess (PMA)) is considered rare and is rarely reported in the literature. This study focuses on infections of the pharyngeal mucosal space, aiming to provide an elaborate description of the clinical behavior of this category and reporting its differences from other categories of deep neck infections (DNIs). **Methods:** A retrospective study of 743 cases of deep neck infections was conducted in a single tertiary center. Cases of abscesses, confined in the pharyngomucosal space (PMAs), were studied, reporting demographics, clinical features, imaging, bacteriology, and treatment data, and comparisons with other DNIs were made. **Results:** A total of 106 cases of pharyngomucosal space abscesses (PMAs) were reported, representing 14.3% of deep neck infections (DNIs). Dysphagia, odynophagia, and fever were the most common symptoms. Hospitalization with non-surgical drainage was the treatment of choice, resulting in complete recovery with no complications. **Conclusions:** In conclusion, the frequency of PMAs in our series suggests that they may not be as rare as they are considered to be. Symptoms and clinical findings can be similar to those of other DNIs, especially parapharyngeal abscesses, but, overall, PMAs seem to have a milder clinical behavior, highlighting the need for diagnosis and reporting as a separate, recognizable category.

## 1. Introduction

Deep neck infections (DNIs) represent a group of relatively common clinical entities but with potentially life-threatening consequences. The advent of antibiotics has led to improved infection outcomes, with wide-spectrum empirical intravenous antibiotics being initiated until culture and sensitivities are reported and targeted therapy is applied [1,2,3]. Nevertheless, the need for potential surgical intervention always comes into consideration. Clinicians are routinely asked to make a call about surgical drainage, deciding on proper timing and surgical access, evaluating the anatomical position of a potential abscess, based on clinical findings and imaging studies.

**Figure 1:**
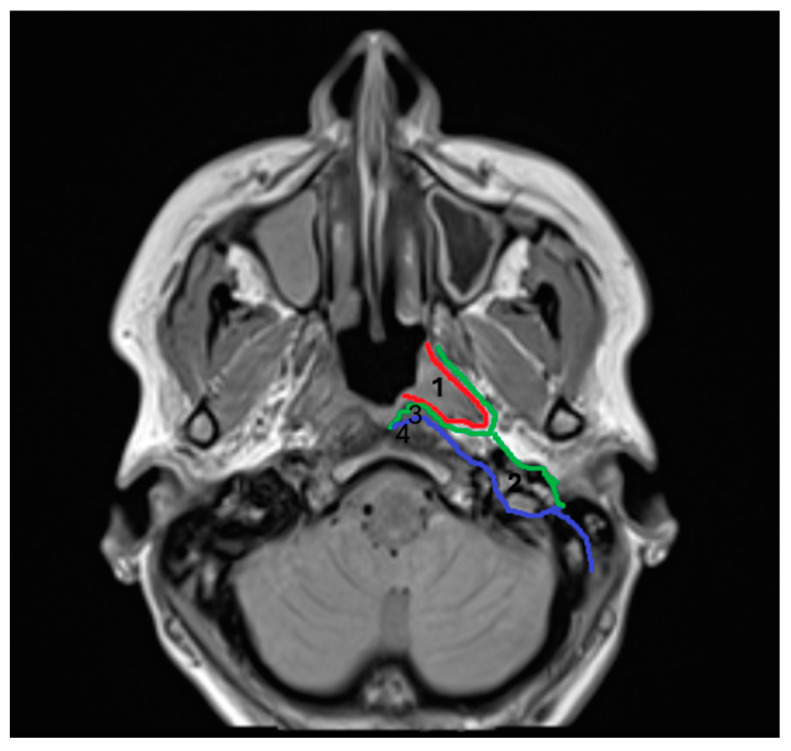
1. Pharyngomucosal space (PMS), 2. parapharyngeal space (PPS), 3. retropharyngeal space (RPS), and 4. prevertebral space (PVS). Red line: pharyngobasilar fascia; green line: middle layer of deep cervical fascia; and blue line: deep layer of deep cervical fascia.

Sound knowledge of pertinent surgical anatomy is of paramount importance in order to access all involved neck spaces [1,2]. The pharyngeal mucosal space (PMS) has been described as the most superficial neck space, lying deep in the pharyngeal mucosa, anteromedially to the parapharyngeal space, and directly anterior to the retropharyngeal space. Its clinical significance has not been recognized until recently, through cross-sectional imaging studies. Anatomically, it represents the area of the nasopharynx and oropharynx on the inner side of the buccopharyngeal fascia. It is located between the pharyngeal mucosa (superficially) and the investing layer of the deep cervical fascia (pharyngobasilar fascia) (deep). The latter surrounds the pharyngeal constrictors. It extends from the skull base to the lower cricoid border, involving the naso-oro-hypopharynx. It is bordered by the superior constrictor muscles’ aponeurosis superiorly, where it merges with the middle layer of deep cervical fascia (Figure 1). PMS consists of five layers, forming the pharyngeal wall. From the internal to the external layers, they include the following: 1. mucosal membrane; 2. submucosa (containing loose areolar tissue, which facilitates deglutition, lymphoid tissue, and minor salivary glands); 3. dense pharyngobasilar fascia; 4. superior constrictor muscles of the pharynx; and 5. buccopharyngeal fascia (outer lining of the space). Dense connective tissue is present only on the deep surface of this layer (deep cervical fascia) [4,5,6,7,8]. Some studies suggest that the peritonsillar space is virtually part of the PMS [4,5,6]; however, more recent studies define them as separate spaces [8].

**Figure 2:**
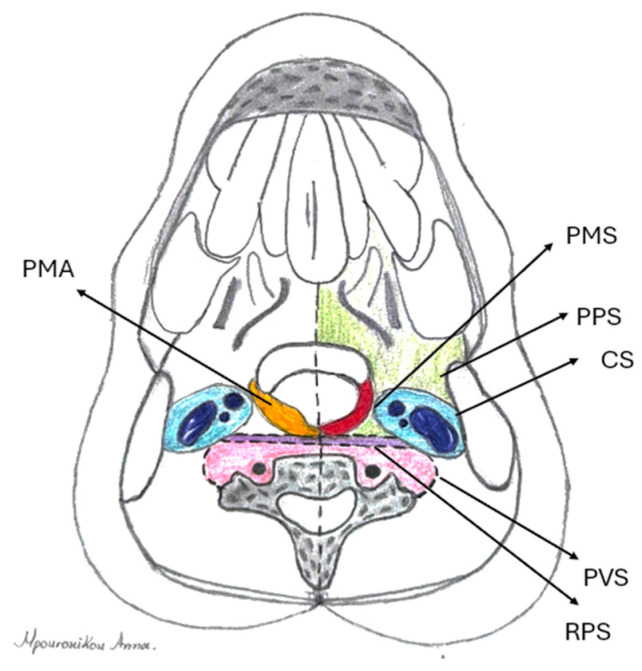
Anatomical relations of pharyngomucosal space (PMS) (red): parapharyngeal space (PPS) (green), carotid space (CS) (blue), prevertebral space (PVS) (purple), and retropharyngeal space (RPS) (pink). Location of pharyngomucosal abscess (PMA) (orange) superficially, in the pharyngeal lumen.

PMS pathology includes neoplasms and congenital and inflammatory lesions [4,5,6,7], while there is a lack of studies on pharyngeal mucosal space abscesses (PMAs) [7]. The isolated collection of pus in the PMS is considered rare; however, the anatomical borders of the PMS suggest a potentially clear clinical distinction between parapharyngeal and peritonsillar abscesses, based on endoscopic and radiologic manifestations (Figure 2) Abscesses confined in the peritonsillar space present with the typical bulging of the peritonsillar space, whereas parapharyngeal abscesses involve neck swelling and diffuse edema of the lateral pharyngeal wall. The observation of inflammation and pus collection, isolated within the lateral pharyngeal wall, has led us to the conclusion that PMAs can be described and treated as a separate category of DNIs.

The purpose of this study was to present our experience and highlight the clinical behavior of abscesses constrained in the PMS, in comparison to other DNIs.

## 2. Materials and Methods

All necessary approvals were obtained, and all participants’ data were handled according to the University Hospital of Larissa Scientific Committee’s regulations, as well as Helsinki and HIPAA regulations.

A retrospective study of 743 consecutive DNIs treated over the period January 2011–December 2019 was conducted. STROBE guidelines were followed. Diagnosis was suspected based on clinical history and findings and confirmed by imaging. The data of patients with an abscess contained only in the PMS, including their demographics, clinical information, bacteriology, and treatment, were exported and analyzed. PMA cases with pus extension to the peritonsillar or any other neck space were excluded. The means were reported with SDs. Χ^2^ test and t-test were used for comparisons; *p* < 0.05 was considered significant. Data analysis was performed with SPSS 20 software (IBM, Chicago, IL, USA).

## 3. Results

**Figure 3:**
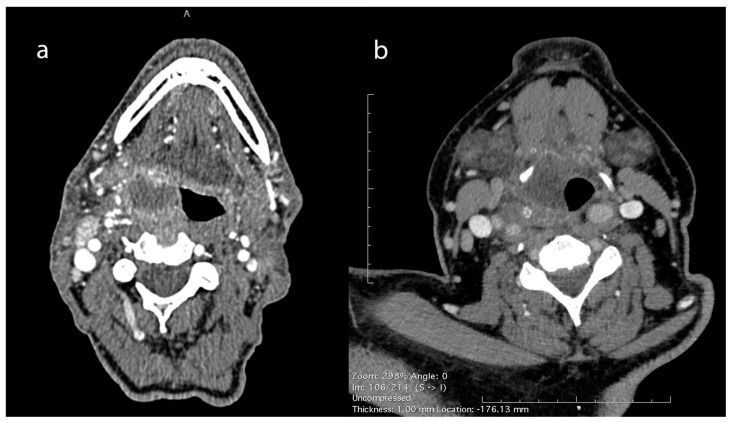
CT scan with contrast of pharyngeal mucosal space abscesses (PMAs). (**a**) Right-side PMA protruding into the pharyngeal lumen, located towards the lateral pharyngeal wall, just under the pharyngeal mucosa and medial to the pharyngeal constrictors, away from the ipsilateral carotid artery and the internal jugular vein. (**b**) Right-side PMA protruding into the pharyngeal lumen and passing medially to the hyoid bone, away from the ipsilateral carotid artery and the internal jugular vein.

PMA diagnosis was the second most common diagnosis, following peritonsillar abscesses, involving 106 adults (female/male ratio: 21/85, mean age: 51 ± 14.1 years). (Table 1). Associated comorbidities included hypertension (*n* = 22), dyslipidemia (*n* = 20), heart disease (*n* = 8), thyroid disease (*n* = 8), diabetes mellitus (*n* = 7), asthma (*n* = 5), anemia (*n* = 3), renal insufficiency (*n* = 1), and rheumatoid arthritis (*n* = 1). Eight patients had a history of tonsillectomy. CT scans with contrast were conducted to confirm the diagnosis (Figure 3).

The symptoms of patients with PMAs are summarized on Table 2. The most commonly reported symptoms were dysphagia, odynophagia, and fever. The mean onset of symptoms was 3.2 ± 1.7 days prior to admission. Trismus and neck swelling were significantly lower in PMAs compared to non-PMA DNIs (*p* < 0.01), while dysphagia was significantly higher (*p* < 0.01). No association with teeth infections was noted in PMAs.

**Figure 4:**
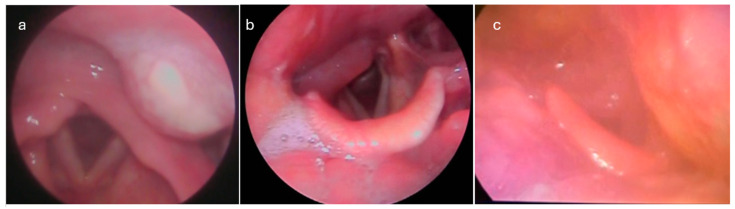
Endoscopic findings in PMA cases. One-sided lateral pharyngeal wall edema (**a**–**c**) and arytenoid edema (**b**) were the common findings during endoscopy.

Table 3 tabulates the summary of patients with PMAs’ physical examination findings. One-sided lateral pharyngeal wall edema was invariable; pyriform sinus, vallecula, uvula, peritonsillar swelling, and unilaterally enlarged tonsil were noticed in 41%, 27%, 27%, 26%, and 18% of patients, respectively, while nasopharyngeal edema and adenoiditis were found in three patients (Figure 4). Tenderness during palpation of the larynx was noted in all patients. The most common laryngeal endoscopic finding was arytenoid edema (20%), whereas the glottic and subglottic areas were spared. Neck swelling was present in 25% of patients.

The mean WBC count and CRP values upon admission were 14.5 ± 5.11 K/μL (reference range, 4–10.8 K/μL) and 13.4 ± 10.1 (reference range, 0–0.5 mg/dL), respectively. Samples for microbiological analysis were obtained in 61 patients; the remaining 45 samples were not processed due to insufficient purulent material. Cultures yielded 23 positive results (Table 4). All isolated bacteria species were aerobic; the commonest were *Streptococcus pyogenes* (52%) and *Staphylococcus aureus* (30%). None of the positive cultures involved multiple bacterial species. Before admission, 37 patients had been under antibiotics and 6 under steroids based on GP recommendations.

In-hospital empirical therapy included IV ampicillin–sulbactam combined with metronidazole or clindamycin. Spontaneous drainage was noted in 52 (49%) patients, transoral drainage under local anesthetic in 47 (44%), and aspiration in 7 (7%). The mean time until spontaneous drainage was 1.8 ± 0.8 days from admission. No further surgical intervention was required, and no major adverse event was noted in any patient, while the mean hospital stay was 4.2 ± 1.8 days.

## 4. Discussion

Up until now, there has been little evidence in the literature about the definition and management of PMA infections. We found only one study (PubMed search) reporting four PMA cases [7]. The term “pharyngeal wall abscess” has also been sporadically used in the literature [9]; thus, no specific description is available. Another study reports four cases of “inferior pole peritonsilar abscesses”, the description of which falls within the spectrum of PMAs [10]. However, in our series, PMAs represent 14,3% of DNIs, suggesting that they are not uncommon, and in “real-world” practice, they may be underdiagnosed or misdiagnosed as parapharyngeal abscesses. Although clinical findings can be similar, major differences were reported in our study concerning etiology, severity of symptoms, and progression of the disease.

PMAs are supposed to be mainly associated with pharyngeal mucosa infections, while infections of the teeth, major salivary glands, or other foci lateral to pharyngeal constrictors rarely result in the formation of PMAs [7]. Our results support this, since none of our cases had an odontogenic or salivary source of infection. As for the clinical features, symptoms of PMAs are overall similar to those of DNIs. Dysphagia was reported consistently by all patients, followed by odynophagia and sore throat. Skoulakis et al. [7] did not report trismus and neck swelling in their cases. In our series, mild trismus was noticed in 21% of patients, but this finding was significantly lower than other DNIs. Neck swelling, secondary to neck lymphadenitis, even though significantly lower than other DNIs, was recorded in 25% of patients. The rest of PMA symptoms were not significantly different from other DNIs, however milder in severity. Fever was reported in half of our patients, whilst the mean WBC and CRP values were raised.

Lateral pharyngeal wall edema was a consistent endoscopic finding in all cases, while, in some of them, edema in other oropharyngeal parts coexisted. In 43 (41%) patients with abscess extension to the hypopharyngeal part of the PMA, edema was extended to the pyriform sinus, while in those where pus reached the most inferior part of the PMS, arytenoid or aryepiglottic fold edema coexisted. No other findings from laryngeal endoscopy were recorded, while follow-up of the five cases with epiglottic edema and the two cases with true vocal cord edema revealed the presence of epiglottic retention cysts and Reinke edema, respectively. Tenderness during palpation of the larynx (bilateral movement) was also a constant finding in all patients. Similar findings were reported by Skoulakis et al. [7].

**Figure 5:**
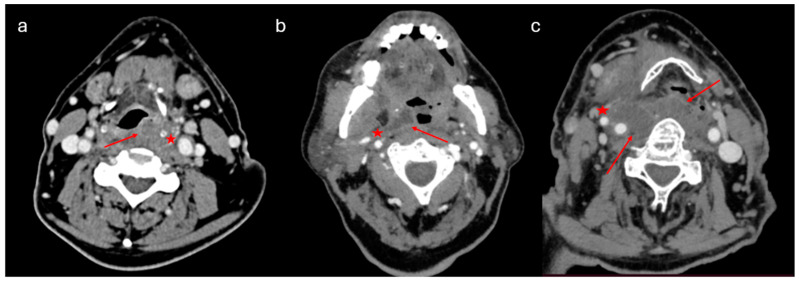
CT images of different categories of DNIs: (**a**) left-side PMA protruding into the pharyngeal lumen, passing medially to the hyoid bone; (**b**) right-side peritonsillar abscess, extending to the parapharyngeal space towards the right carotid artery and jugular vein; and (**c**) extended right-side parapharyngeal and retropharyngeal abscess. Arrows and stars indicate the margins of the abscess.

Although clinical findings are of the greatest importance in the suspicion of a PMA, a CT scan with contrast is the “gold standard” to set the diagnosis [7] (Figure 3 and Figure 5). Abscess protrusion into the pharyngeal lumen may give the false impression of a parapharyngeal abscess; however, the PMS is located in the lateral pharyngeal wall, between the pharyngeal mucosa and the pharyngeal constrictors, extending under the hyoid bone and passing medially to it. On the contrary, other spaces extend laterally to the hyoid bone (e.g., carotid space), whereas, for others (e.g., parapharyngeal space), it represents their lower border. Ultrasonography cannot identify the PMS adequately, while MRI may also be of use in selected cases [7]. The high diagnostic accuracy of MRI can be utilized in order to specify the anatomic relation of the abscess with the pharyngeal constrictors, differentiating between PMAs and deeper-extending abscesses [11]. However, the practical need for imaging and the cost-effectiveness can be controversial, as a significant number of cases can resolve rapidly. Nevertheless, unless strongly contraindicated, imaging is highly recommended, so that deeper counterpart involvement may be excluded, especially in cases with severe symptoms and clinical findings.

*Streptococcus pyogenes* and *Staphylococcus aureus* were the most common bacteria species in our series. The microbiology of PMAs seems to be similar to other DNIs and strongly correlated to pharyngeal microflora [3,7]. However, we only had a small number of positive cultures, so, on these grounds, safe and generalized conclusions cannot be made. Empirical therapy with IV-ampicillin–sulbactam combined with metronidazole or clindamycin proved to be effective in our practice.

Skoulakis et al. suggest that “*the abscess, as a rule, is drained spontaneously*” [7]. This clinical feature is of major importance, differentiating significantly the clinical behavior of PMAs from other deep neck infections. In our series, spontaneous drainage was noted in 49% of patients, and the mean time until spontaneous drainage was 1.8 ± 0.8 days from admission. In all instances, the spontaneous pharyngeal mucosa opening was small, and the flow of pus was slow, often allowing the patient to swallow it. We believe that this was the main reason why no complications related to tracheobronchial pus aspiration were noted in any of these patients. The PMS lies just deep to the pharyngeal mucosa, while dense connective tissue is present only at its deeper border, hence hindering infection from spreading towards deeper spaces and facilitating pus drainage of potential abscesses intraluminally, by virtue of least resistance. Furthermore, the pharyngeal constrictors’ pressure on the abscess during the pharyngeal peristaltic wave facilitates spontaneous drainage through the “vulnerable” mucosa. Transoral drainage under local anesthesia or aspiration may also be considered, in order to speed up recovery and shorten hospital-stay. The lack of established guidelines in the literature, concerning algorithms on the management of DNIs, is possibly due to the heterogeneity of clinical manifestations. However, the basic principle remains the need for hospitalization and surgical drainage, taking into account risk factors for major complications [12]. Thus, the clinical importance of recognizing a PMA can affect the decision-making process when surgery comes into question and can predict the duration of in-hospital treatment, as minimal interventions seem to be adequately effective.

In our series, all PMAs were managed conservatively, with minimal intervention, no major adverse events were noted, and no further surgical interventions were needed. Parapharyngeal abscesses, however, and multi-space abscesses (involving more than one space; the majority including the parapharyngeal space) were treated surgically (63%, 66%, respectively). Additionally, the mean hospital stay in the PMA group was significantly lower (Table 5). Major complications of other DNIs, such as mediastinitis and airway obstruction, are unlikely to occur because of the superficial location of the PMS in the pharyngeal lumen. A dense deep border, constricting the PMS from spreading into the deep layer of the deep cervical fascia, together with spontaneous drainage may be the reason why abscesses constrained in PMS fare better than parapharyngeal and retropharyngeal abscesses, which are associated with high rates of major complications and the need for, sometimes multiple, surgical interventions [13].

### Limitations

Even though our data were withdrawn from a prospectively collected database, the retrospective nature of our study represents its main limitation, along with the fact that the data had been collected at a single center. However, the relatively large number of cases minimizes the bias and provides meaningful results. The existing literature gap regarding this topic limits the theoretical foundations of our study, therefore highlighting the need for further research. Potential multi-center observational studies are required to confirm our results and assess the description of PMAs in the future.

## 5. Conclusions

Our study suggests that PMAs are not as rare as they are considered to be; however, there is a lack of research on abscesses in this space. PMAs have much in common with other DNIs: PMAs’ symptoms and clinical findings are similar to those of other DNIs; however, trismus and neck swelling seem to be significantly lower in PMAs. Projection of the lateral pharyngeal wall represents the most consistent finding, calling for differential diagnosis, mainly between parapharyngeal and PMS abscesses.

Imaging studies can confirm the diagnosis, with CT with contrast being the modality of choice. Overall, PMAs seem to be less dangerous than their deep-seated counterparts, since their superficial location renders them amenable to spontaneous drainage, aspiration, incision, and intraoral drainage, obviating expansion into deeper structures.

In conclusion, we believe that lesions of the pharyngeal mucosal space represent a group of cervical lesions with special clinical and radiological characteristics that require diverse methods of treatment. Familiarization with the anatomy of the pharyngeal mucosal space and the clinical course of its inflammatory lesions can lead to optimal management of these not-so-rare cases, potentially sparing patients from unnecessary imaging and surgical intervention. Our assessment is that, as a rule, the diversity of this space is not adequately acknowledged by clinicians; thus, more data reporting and further research are required in order to ensure that all cases of inflammations of this space are recognized and treated correctly.

## Figures and Tables

**Table 1:** Demographics and involved neck spaces of 743 patients with deep neck infections (DNIs).

Space Involved			Female/Male	Mean Age ± SD
	*n* = 743	%	243/500	39.55 ± 17.18
Peritonsillar	527	70.9	179/348	35.46 ± 14.9
Pharyngomucosal	106	14.3	21/85	51 ±14.1
Submandibular	46	6.2	17/29	45.39 ± 21.3
Parapharyngeal	11	1.5	4/7	43.81 ± 28.37
Retropharyngeal	8	1.1	5/3	45.5 ± 28.3
Ludwig’s angina	6	0.8	3/3	64.83 ± 14.91
Masticator	3	0.4	1/2	34.66 ± 6.8
Parotid	3	0.4	1/2	45 ± 19.69
Visceral	1	0.1	1/0	60
Multi-space (>1 of the above)	32	4.3	11/21	52.34 ± 16.57

**Table 2:** Summary of the patients presenting symptoms with pharyngeal mucosal space abscesses (PMA) versus non-pharyngeal mucosal space deep neck infections (DNIs).

	PMA		Non-PMA DNIs		
Symptoms	*n* = 106	%	*n* = 637	%	*p*
dysphagia	106	100.00	591	92.78	0.004 *
odynophagia/sore throat	63	59.43	348	54.63	0.357
fever	53	50.00	350	54.95	0.344
neck swelling	26	24.53	545	85.56	<0.001 *
trismus	22	20.75	285	44.74	<0.001 *
“hot potato” voice	18	16.98	116	18.21	0.815
drooling	10	9.43	47	7.38	0.462
breathing discomfort	3	2.83	14	2.20	0.604
ear pain	2	1.89	9	1.41	0.708
malaise	1	0.94	31	4.87	0.65
odontalgia	-	-	43	6.75	0.006 *

* Significant difference (*p* < 0.05).

**Table 3:** Summary of physical examination findings upon admission of patients with pharyngeal mucosal space abscesses.

	*n* = 106	%
**Oral Cavity**		
odontogenic source of infection	0	0
poor dentition	46	43.4
no clinically noticeable findings	60	56.60
**Oropharynx**		
lateral pharyngeal wall edema	106	100
vallecula edema	29	27.36
uvula edema	29	27.36
peritonsillar edema	28	26.42
unilaterally enlarged tonsil	19	17.92
soft palate edema	10	9.43
posterior pillar edema	6	5.66
tonsillar exudate	4	3.77
anterior pillar edema	3	2.83
**Hypopharynx**		
pyriform sinus edema	43	40.57
**Nasopharynx**		
edema/adenoiditis	3	2.83
no clinically noticeable findings	103	97.17
**Larynx**		
tenderness during palpation	106	100
**Supraglottic**		
arytenoid cartilage edema	21	19.81
aryepiglottic fold edema	9	8.49
edema of the laryngeal surface of the epiglottis (retention cyst *)	5	4.72
no clinically noticeable findings	81	76.42
**Glottic**		
true vocal cord edema (Reinke edema *)	2	1.89
no clinically noticeable findings	104	98.11
**Subglottic**		
no clinically noticeable findings	106	100
**Neck**		
cervical lymph nodes	26	24.53
- painless	13	12.26
- tenderness	13	12.26
no clinically noticeable findings	80	75.47
**Nasal Cavities**		
clear secretions	14	13.21
purulent secretions	1	0.94
no clinically noticeable findings	91	85.85
**Salivary Glands/Cranial Nerves/Ears**		
no clinically noticeable findings	106	100

* Follow-up finding.

**Table 4:** Distribution of the species isolated from the positive cultures (*n* = 23) of pharyngeal mucosal space abscesses.

	*n*	(%)
**Aerobic Bacteria**		
**a.** **Gram-positive**	**21**	**91.3**
1. *Streptococcus pyogenes*	12	52.2
2. *Staphylococcus aureus*	7	30.4
3. *Streptococcus group C*	1	4.3
4. *Streptococcus anginosus*	1	4.3
**b.** **Gram-negative**	**2**	**8.7**
1. *Pseudomonas aeruginosa*	2	8.7

**Table 5:** Surgical treatment and duration of hospital stay.

Space Involved	Total	Surgical Drainage		Mean Hospital Stay ± SD
		*n*	%	
Pharyngomucosal	106	0	0	4.2 ± 1.8
Parapharyngeal	11	7	63.6	9.88 ± 4.51 (*p* < 0.0001 *)
Multi-space (incl. parapharyngeal, submandibular, retropharyngeal, peritonsillar, masticator, visceral)	32	21	65.6	12 ± 8.8 (*p* < 0.0001 **)

* Unpaired *t*-test, t = 8.2473, df = 115, ** Unpaired *t*-test, t = 8.6141, df = 136.

## Data Availability

The data presented in this study are available on request from the corresponding author due to privacy reasons.

## References

[1] Christian J.M., Goddard A.C., Gillespie M.B. (2015). Deep Neck and Odontogenic Infections. Cummings Otolaryngology—Head and Neck Surgery.

[2] Chan Y., Goddard J.C. (2016). Neck Spaces and Fascial Planes. KJ Lee’s Essential Otolaryngology.

[3] Beka D., Lachanas V.A., Doumas S., Xytsas S., Kanatas A., Petinaki E., Skoulakis C. (2019). Microorganisms involved in deep neck infection (DNIs) in Greece: Detection, identification and susceptibility to antimicrobials. BMC Infect. Dis..

[4] Parker G.D., Harnsberger H.R., Jacobs J.M. (1990). The pharyngeal mucosal space. Semin. Ultrasound CT MR.

[5] Harnsberger H.R., Harnsberger H.R. (1995). The parapharyngeal and pharyngeal mucosal space. Handbook of Head and Neck Imaging.

[6] Mafee M.F., Valvasorri G.E., Becker M. (1995). Imaging of the Head and Neck.

[7] Skoulakis C.E., Papadakis C.E., Bizakis J.G., Nikolidakis A.A., Manios A.G., Helidonis E.S. (2003). Abscess of the pharyngeal mucosal space—An unusual location. J. Otolaryngol..

[8] Warshafsky D., Goldenberg D., Kanekar S.G. (2012). Imaging anatomy of deep neck spaces. Otolaryngol. Clin. N. Am..

[9] Khoury M., Dong S.X., Alsaffar H., Johnson-Obaseki S., Caulley L. (2022). Isolated oropharyngeal abscess with hypopharyngeal extension recurring 12 years after initial surgical management: A case report and review of the literature. SAGE Open Med. Case Rep..

[10] Licameli G.R., Grillone G.A. (1998). Inferior pole peritonsillar abscess. Otolaryngol. Head Neck Surg..

[11] Vierula J.P., Nurminen J., Jussila V., Nyman M., Heikkinen J., Pape B., Sorvettula K., Mattila K., Hirvonen J. (2023). Diagnostic performance of short noncontrast biparametric 3-T MRI for tonsillar infections: Comparison with a full protocol including contrast-enhanced sequences. Eur. Radiol. Exp..

[12] Sheikh Z., Yu B., Heywood E., Quraishi N., Quraishi S. (2023). The assessment and management of deep neck space infections in adults: A systematic review and qualitative evidence synthesis. Clin. Otolaryngol..

[13] Loperfido A., Stasolla A., Giorgione C., Mammarella F., Celebrini A., Acquaviva G., Bellocchi G. (2023). Management of Deep Neck Space Infections: A Large Tertiary Center Experience. Cureus.

